# Biologic Augmentation of the Ulnar Collateral Ligament in the Elbow of a Professional Baseball Pitcher

**DOI:** 10.1155/2015/130157

**Published:** 2015-07-09

**Authors:** James K. Hoffman, Nicole M. Protzman, Amit D. Malhotra

**Affiliations:** ^1^Department of Orthopedics, Coordinated Health, 2775 Schoenersville Road, Bethlehem, PA 18017, USA; ^2^Clinical Education and Research Department, Coordinated Health, 3435 Winchester Road, Allentown, PA 18104, USA; ^3^Imaging Department, Coordinated Health, 2300 Highland Avenue, Bethlehem, PA 18020, USA

## Abstract

Tears of the ulnar collateral ligament (UCL) of the elbow are common injuries in overhead athletes. Although surgical reconstruction of the UCL has improved outcomes, not all athletes return to their previous level of competition and when this goal is achieved, the time required averages one to two years. Therefore, additional techniques are needed to further improve return to play and the rate of return to play in overhead athletes. A construct comprising a dermal allograft, platelet rich plasma (PRP), and mesenchymal stem cells (MSCs) has been shown to successfully improve healing in the rotator cuff. Given the promising provisional findings, we postulated that this construct could also improve healing if applied to the UCL. Therefore, the purpose of the present report was to examine the feasibility of utilizing a dermal allograft, PRP, and MSC construct to augment UCL reconstruction in a professional baseball pitcher. No complications were encountered. Although limited to minimal follow-up, the patient has demonstrated excellent progress and has returned to activity.

## 1. Introduction

The anterior band of the ulnar collateral ligament (UCL) provides stability to valgus stress at the elbow [1, 2]. During overhead activities, this ligament is subjected to extreme tension, which places the overhead-throwing athlete at significant risk for injury. Given the repetitive, high-tensile load placed on the elbow during throwing, baseball pitchers are especially susceptible to UCL insufficiency [3–5]. Acute or chronic UCL disruption and attenuation often result in medial elbow pain, valgus instability, neurologic compromise, and impaired throwing performance. After exhaustion of conservative treatment options, if the athlete wishes to return to throwing activities, surgical repair is often required.

Injury to the UCL was once viewed as the end of a baseball pitcher's career. In 1974, however, Dr. Frank Jobe and his colleagues performed the first successful UCL reconstruction on the Los Angeles Dodgers pitcher, Tommy John, coining the term “Tommy John Surgery.” Since its initial introduction, UCL reconstruction has evolved, and its success has drastically improved.

In the initial report by the pioneering surgeon, 63% of overhead athletes returned to a preinjury level of participation [5]. In a recent examination of major league baseball players, this value increased to 97% [6]. Despite these noteworthy improvements, the time required for the athlete to return to the previous level of competition averages one to two years, but not all athletes achieve this goal [6–8]. Therefore, techniques capable of improving UCL repair and accelerating the healing cascade are warranted.

A construct comprising a dermal allograft, platelet-rich plasma (PRP), and mesenchymal stem cells (MSCs) was previously used to augment a rotator cuff repair [9, 10]. It was shown to successfully promote collagen fiber reorganization, remodeling, and incorporation into the native tissue with noted angiogenesis and neural infiltration [9]. Given these promising provisional findings, the senior author postulated that this construct could also improve healing if applied to the UCL. To date, no report has described the application of MSCs to augment UCL repair or reconstruction. Therefore, the purpose of the present report was to examine the feasibility of utilizing a dermal allograft, PRP, and MSC construct to augment UCL reconstruction in a professional baseball pitcher.

## 2. Case Presentation

In September 2013, a 25-year-old professional baseball pitcher presented with left elbow pain. He noted that his pain had been ongoing since June 2012, but over the prior three months, he developed swelling and numbness that radiated to his fourth and fifth phalanges. In May 2013, after three orthopedic upper extremity evaluations, the pitcher discontinued his participation in baseball.

### 2.1. Physical Examination

Examination of the left elbow demonstrated no visible abnormalities, no erythema, no olecranon swelling, no effusion, no deformity, no warmth, and no ecchymosis. Palpation of the left elbow demonstrated medial epicondyle tenderness but did not demonstrate radial head tenderness, lateral epicondyle tenderness, biceps insertion tenderness, triceps insertion tenderness, or any tenderness consistent with olecranon bursitis. A positive ulnar nerve Tinel's sign was appreciated. Abnormal muscle tone was noted about the left elbow. The patient demonstrated painless motion in all directions, no crepitus with motion, and 5/5 motor strength. Joint laxity of the left elbow was noted with valgus stress at 30° of flexion. No rotational instability was appreciated.

### 2.2. Tests and Results

In July 2012, a magnetic resonance evaluation was performed. Posteromedial osteophytes between the opposing olecranon and trochlear articular surfaces suggested the possibility of valgus extension overload syndrome. A small ossicle was seen at the humeral attachment of the UCL with adjacent soft tissue edema, likely secondary to chronic avulsion injury.

**Figure 1:**
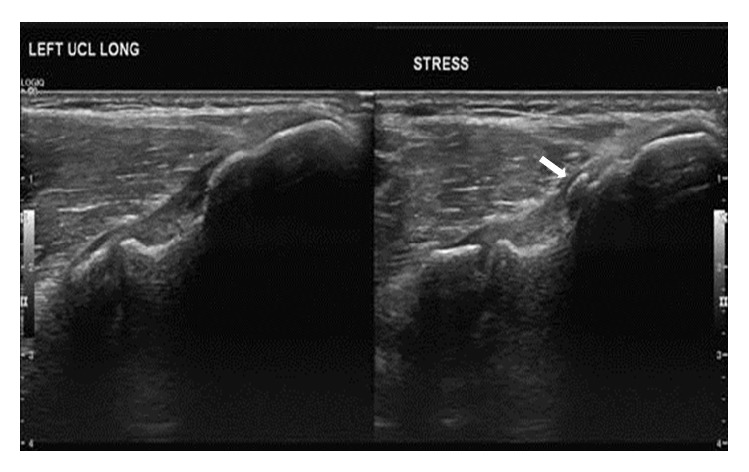
Preoperative ultrasound. Images demonstrate mild thickening of the proximal fibers of the proximal ulnar collateral ligament (medial epicondyle is to the right of image, and ulna is to the left of the image), likely sequela of prior or chronic repetitive low grade injury. Small area of heterotopic ossification embedded within the proximal fibers of the ulnar collateral ligament (arrow). Dynamic stress images demonstrate mild “gapping” of the medial compartment (2 mm) suggestive of laxity.

In July 2013, an EMG of the left upper extremity was performed. It revealed left ulnar compression neuropathy. Then in September 2013, a dynamic ultrasound was ordered. The dynamic ultrasound revealed thickening of the ulnar nerve within the cubital tunnel, with no signs of subluxation (Figure 1).

Considering the clinical presentation and diagnostic findings, the patient was diagnosed with UCL instability and ulnar nerve neuritis. Per the orthopedic surgeon's evaluation, the patient was deemed eligible for an UCL reconstruction with a dermal allograft, PRP, and MSC construct and ulnar nerve decompression. He elected to undergo surgery. The surgery was scheduled for October 2013.

### 2.3. Augmentation of Ulnar Collateral Ligament Reconstruction

**Figure 2:**
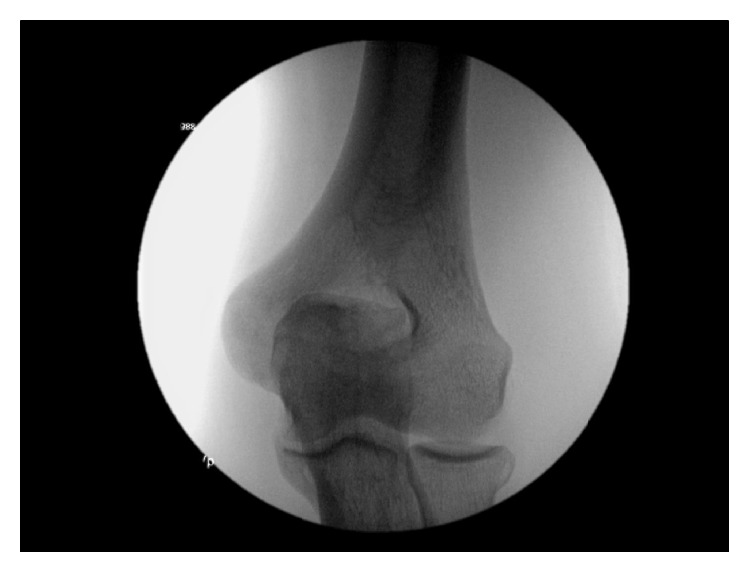
Intraoperative radiograph. Intraoperative stress view of the left elbow demonstrating valgus laxity.

The patient was placed in a supine position on the operating room table. Stress views of the left elbow were obtained at 70° of elbow flexion (Figure 2). Under valgus stress, there was more than a 2 mm gap between the humerus and olecranon.

**Figure 3:**
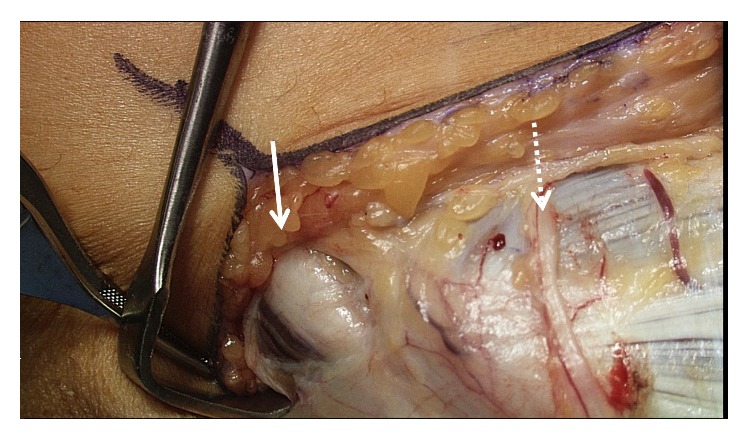
The internervous plane. The ulnar nerve (solid arrow) and medial antebrachial cutaneous nerves (dotted arrow) were identified and protected.

**Figure 4:**
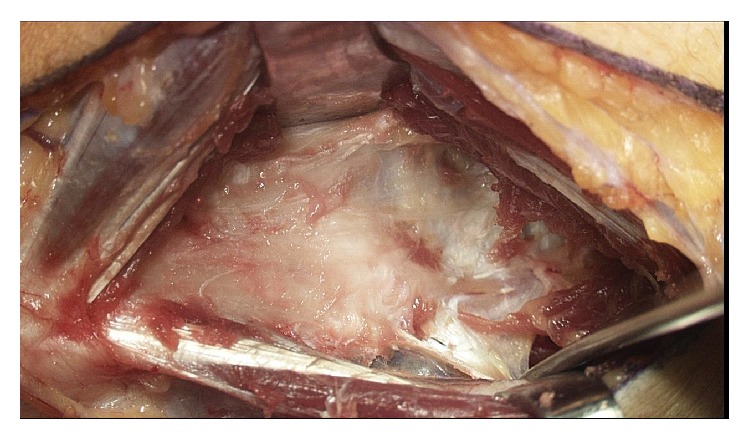
Exposure of medial elbow through internervous plane. Note that the flexor mass and flexor carpi ulnaris were not detached.

An assessment and comparison was made with the right elbow. There was no instability present on the right elbow. An incision was made over the medial epicondyle and carried down to the subcutaneous tissue. Medial antebrachial nerve branches were identified and protected. The pronator, flexor mass, and flexor carpi ulnaris were identified. An internervous plane was utilized between the flexor carpi ulnaris and the flexor mass (Figure 3). This plane was separated to gain exposure to the UCL (Figure 4). Neither the flexor mass nor the flexor carpi ulnaris were detached.

The ulnar nerve was identified and decompressed. An intraoperative nerve stimulator was utilized (0.5 mV) to verify proper functioning of the ulnar nerve. The nerve was confirmed to be intact with proper function both before and after reconstruction of the UCL.

The superior, oblique, and posterior aspects of the UCL were identified. As previously identified with advanced radiography, there were two bone spurs. One was located on the distal humerus and the other on the olecranon. A longitudinal incision was made in the capsule and ulnar collateral ligament. The bony prominences were removed with a rongeur and bone bur.

The anterior superior band of the UCL was identified under valgus stress and was found to be incompetent. The UCL was repaired with a dermal allograft (Musculoskeletal Transplant Foundation, Edison, NJ, USA) rehydrated in PRP and MSCs (Ovation, Osiris Therapeutics, Columbia, MD). The dermal allograft was reduced in size to fit the UCL and medial aspect of the joint.

**Figure 5:**
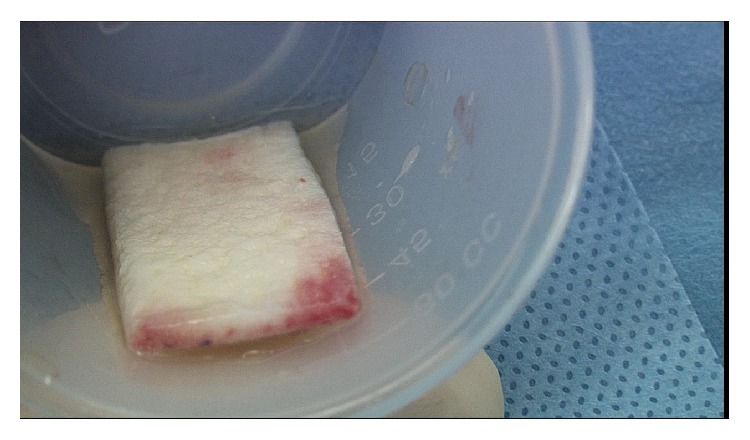
Dermal allograft. The dermal allograft was reconstituted with platelet-rich plasma and mesenchymal stem cells.

**Figure 6:**
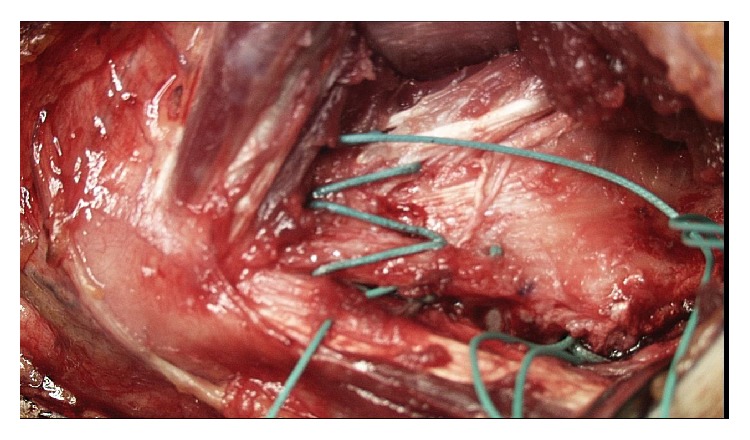
Preapplication of biologic construct. The bed was prepared for the application of the biologic construct.

**Figure 7:**
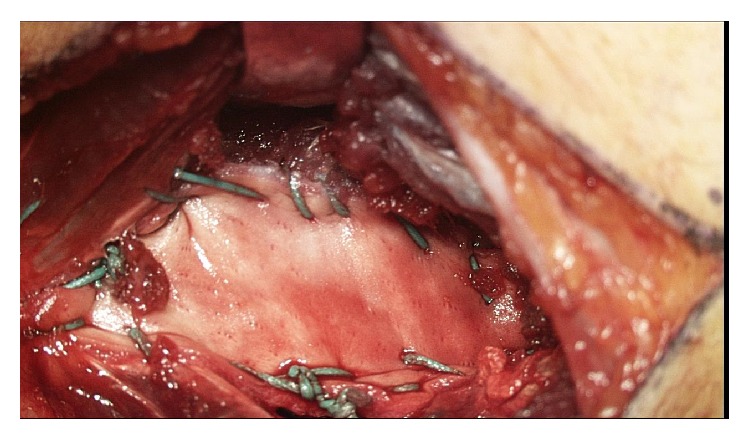
Biologic construct application. The dermal allograft application for the reconstruction of the ulnar collateral ligament.

One milliliter of stem cells was thawed in room temperature saline solution. Blood, which had been drawn from the patient preoperatively, was spun down to form 5 mL of PRP (Cascade Autologous Platelet-Rich Plasma, Musculoskeletal Transplant Foundation, Edison, NJ). The graft was reconstituted in PRP and MSCs (Figure 5). Anchor sutures were placed on the humeral and ulnar side to anchor the graft directly on the remaining UCL (Figure 6). Positioning was verified under fluoroscopy. The reconstituted graft was then advanced and tied with permanent sutures from the anchors. Horizontal mattress sutures were placed proximally and distally. The graft was then sutured centrally with horizontal mattress sutures, which were placed through the ulnar collateral ligament and capsule. Complete reapproximation of the dermal allograft to the ligament was achieved under proper physiologic tension (Figure 7). The remaining PRP was inserted into the region under the flexor mass and flexor carpi ulnaris. The flexor mass was reapproximated to the flexor carpi ulnaris. The medial antebrachial cutaneous nerves were maintained. The upper extremity was then placed in an articulated brace with the elbow at 90 degrees. Postoperatively, an upper extremity nerve block was performed.

### 2.4. Outcomes

The patient was referred to occupational therapy for range of motion for the hand and wrist. The patient remained in an elbow brace for a total of six weeks. For the first three weeks, the brace was locked at 90 degrees of flexion. The first two weeks of therapy focused on active range of motion for the wrist, forearm, and digits, isometric wrist exercises were performed, and wrist and hand conditioning tasks were initiated. Three weeks following the repair, the patient began gravity assisted range of motion exercises at the elbow and strengthening of the hand and wrist. At four weeks postoperatively, active elbow flexion and extension were initiated. Elbow exercises were progressively increased through active, active assisted, and gravity assisted range of motion. The patient reported initiating strengthening tasks at home at approximately six weeks and progressively increasing the training regimen.

**Figure 8:**
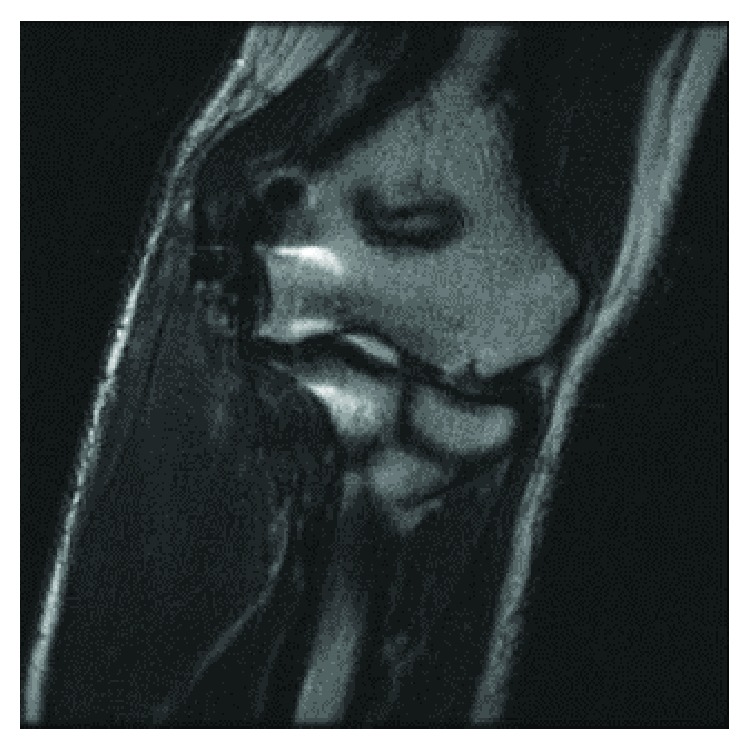
Three-month postoperative radiograph. Coronal T2 weighted images demonstrate low signal intensity fibers extending from the medial epicondyle of the distal humerus to the medial margin/sublime tubercle of the proximal ulnar. No tear, deformity, or retraction of the ulnar collateral ligament reconstruction site.

**Figure 9:**
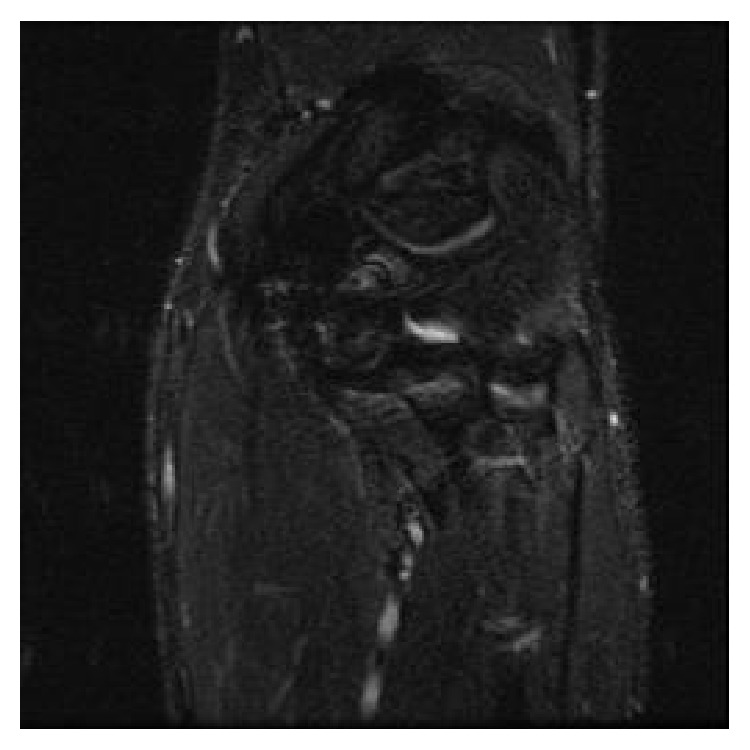
17-month postoperative radiograph. Coronal STIR MRI demonstrates postoperative changes and the medial aspect of the elbow. There are intact low signal intensity fibers extending from the medial epicondyle to the sublime tubercle of the proximal ulna, without retraction or deformity at the reconstruction site.

Three months postoperatively, an MRI was ordered to evaluate the status of the surgical repairs. The MRI revealed an intact dermal allograft with no surrounding inflammatory response (Figure 8). A throwing program was initiated shortly afterward, approximately four months following surgery. A subsequent MRI was ordered 17 months postoperatively, which demonstrated an intact dermal allograft with no retraction or deformity at the reconstruction site (Figure 9).

The patient is currently one year and nine months following the UCL reconstruction. He is doing very well. There is no indication of ulnar neuropathy or instability. The patient had an uncomplicated postoperative course with a successful return to throwing 86 miles per hour.

## 3. Discussion

Ulnar collateral ligament reconstruction consistently permits successful return to play for professional baseball pitchers [6]. In a recent comparative investigation, patients who underwent UCL reconstruction had fewer losses per season and a lower losing percentage, compared with demographic-matched controls [6]. Even so, there is room for improvement.

To our knowledge, only one study has examined the use of biologics to augment healing of the UCL [11]. Podesta and colleagues examined the use of a PRP injection to promote healing of partial UCL tears in overhead athletes [11]. Encouragingly, 30 of 34 overhead athletes returned to their previous level of competition at an average of 12 weeks with resultant improvements in the Kerlan-Jobe Orthopaedic Clinic Shoulder and Elbow score, Disabilities of the Arm, Shoulder, and Hand score, and decreases in the medial elbow joint space [11]. These results suggest that PRP injections may effectively treat partial tears of the UCL, while also demonstrating the promising role of biologics in the repair of the UCL of the elbow.

Similar to application in the rotator cuff [9, 10], in the present case, an acellular dermal allograft was used as a vehicle to deliver PRP and MSCs to the damaged tissue. The dermal allograft was fixated directly onto the UCL, which increased the structural integrity of the repair. Given its architecture, the acellular graft also provided a framework for cellular infiltration and three-dimensional ingrowth. Prior to implantation, the allograft was reconstituted in a mixture of PRP and MSCs. When activated, PRP releases a directly proportional quantity of growth factors [12]. These bioactive molecules are capable of accelerating the healing cascade through tissue repair and regeneration. Recent research has suggested that growth factors may provide the necessary cellular and molecular signals to optimize the function of MSCs [13]. Through direct differentiation and paracrine signaling, MSCs demonstrate dynamic reparative properties. These nonhematopoietic, multipotent cells are capable of differentiating into multiple tissue-forming cell lineages, such as tenocytes, chondrocytes, and osteoblasts [14–16], and through paracrine signaling, they control the local cellular environment by releasing biologically active molecules, such as growth factors. Given the potential to stimulate healing and tissue repair, MSCs have gained increasing popularity. Although the evidence is preliminary and limited to case reports [9, 10], this trifecta of biologics appears capable of creating an optimal healing environment.

In the present case report, the patient demonstrated improvements in pain and function and returned to pitching at 86 miles per hour. These provisional findings support the use of a dermal allograft, PRP, and MSCs to augment the UCL reconstruction. However, the successful outcome cannot be solely attributed to the biologic construct. The postoperative rehabilitation protocol also plays a crucial role. The physician worked closely with the occupational therapist to design a rehabilitation program in accordance with the temporal parameters of MSC-based healing. During the immediate postoperative period, it is important to select exercises that avoid placing adverse strain on the repair. Therefore, the rehabilitation protocol was progressively staged with passive range of motion, active range of motion, isometric muscle contraction, and strengthening. When the athlete demonstrated adequate progress, a throwing program was initiated.

Tears of the UCL were once career-ending injuries. Over time, techniques evolved and return to athletic participation improved [17]. In the present report, the authors introduced a potential means of augmenting UCL repair. Additional research studies with higher levels of evidence are necessary to compare conventional UCL reconstruction with our augmentation technique to determine if significant improvements in return to play and the rate of return to play are achieved in overhead athletes.
